# Impact of Left Ventricular Diastolic Dysfunction and Biomarkers on Pulmonary Hypertension in Patients with Severe Aortic Stenosis

**DOI:** 10.3390/medicina54040063

**Published:** 2018-09-04

**Authors:** Birutė Gumauskienė, Aušra Krivickienė, Regina Jonkaitienė, Jolanta Justina Vaškelytė, Adakrius Siudikas, Eglė Ereminienė

**Affiliations:** 1 Department of Cardiology, Medical Academy, Lithuanian University of Health Sciences, LT-50161 Kaunas, Lithuania; krivickiene.ausra@gmail.com (A.K.); jonkaitiene.regina@gmail.com (R.J.); jvaskelyte@gmail.com (J.J.V.); eglerem@yahoo.com (E.E.); 2 Department of Cardiac, Thoracic and Vascular Surgery, Medical Academy, Lithuanian University of Health Sciences, LT-50161 Kaunas, Lithuania; asiudikas@hotmail.com

**Keywords:** aortic stenosis, pulmonary hypertension, diastolic dysfunction, NT-proBNP, GDF-15

## Abstract

*Background*: Severe aortic stenosis (AS) complicated by pulmonary hypertension (PH) is associated with poor outcomes after surgical aortic valve replacement (AVR). There is still scarce information about predictors of secondary PH in this group of patients. *Objectives*: The aim of this study was to investigate the prognostic impact of biomarkers together with conventional Doppler echocardiographic parameters of left ventricular diastolic function on elevated pulmonary systolic pressure (PSP) in severe AS patients before surgical AVR. *Methods*: Sixty patients with severe isolated AS (aortic valve area <1 cm^2^) underwent echocardiography, N-terminal pro B-type natriuretic peptide (NT-proBNP) and growth differentiation factor-15 (GDF-15) measurements before AVR. PSP, left ventricular ejection fraction (LV EF), parameters of LV diastolic function (E/E’ ratio, mitral valve deceleration time (MV DT) and left atrial (LA) volume) were evaluated. PH was defined as an estimated PSP ≥ 45 mmHg. *Results*: Of the 60 patients, 21.7% with severe isolated AS had PH with PSP ≥ 45 mmHg (58.5 ± 11.2 mmHg). LV EF did not differ between groups and was not related to an elevated PSP (50 ± 8 vs. 49 ± 8%, *p* = 0.58). Parameters of LV diastolic dysfunction (E/E’ ratio > 14 (OR 6.00; 95% CI, 1.41–25.48; *p* = 0.009), MV DT ≤ 177.5 ms (OR 9.31; 95% CI, 2.06–41.14; *p* = 0.001), LA volume > 100 mL (OR 9.70; 95% CI, 1.92–49.03; *p* = 0.002)) and biomarkers (NT-proBNP > 4060 ng/L (OR 12.54; 95% CI, 2.80–55.99; *p* < 0.001) and GDF-15 > 3393 pg/mL (OR 18.33; 95% CI, 2.39–140.39; *p* = 0.001)) were significantly associated with elevated PSP in severe AS. *Conclusions*: Left ventricular diastolic dysfunction and elevated biomarkers levels could predict the development of pulmonary hypertension in patients with severe aortic stenosis. Elevation of biomarkers paired with worsening of LV diastolic dysfunction could help to stratify patients for earlier surgical treatment before the development of pulmonary hypertension.

## 1. Introduction

Degenerative aortic stenosis (AS) is the most common acquired valvular heart disease and its prevalence is expected to increase due to the aging population [1,2]. Pulmonary hypertension (PH) can be found in up to 75% of patients with symptomatic severe AS and represents a marker of more advanced disease and major adverse cardiovascular events, as well as augmented mortality regardless of surgical or interventional treatment [3,4,5,6,7]. To date, the predictors of PH in severe AS remain uncertain.

Several biomarkers, reflecting structural and biological changes of myocardium in response to chronic pressure overload in AS patients, were found to predict presence of symptoms and cardiovascular death or hospitalization in this group of patients [8,9,10]. However, data about the impact of biomarkers on the development of PH in severe AS are lacking. The multi-marker approach, including conventional echocardiographic parameters of left ventricular (LV) systolic and diastolic function, is of great importance to find out the cut-off values for elevated pulmonary pressure in severe AS and could improve the risk stratification in patients for earlier surgical aortic valve replacement (AVR) before the development of PH.

The purpose of our study was to investigate the prognostic impact of biomarkers together with conventional echocardiographic parameters of LV diastolic function on elevated pulmonary systolic pressure (PSP) in severe isolated AS patients.

## 2. Materials and Methods

### 2.1. Study Population 

The prospective study (performed between November 2014 and May 2016) included 60 patients with symptomatic severe AS, defined as an aortic valve area less than 1 cm^2^, who underwent two-dimensional Doppler echocardiographic evaluation, N-terminal pro-brain natriuretic peptide (NT-proBNP) and growth differentiation factor 15 (GDF-15) measurement before AVR. Patients with documented coronary heart disease or chronic pulmonary disease, atrial fibrillation and moderate–severe mitral regurgitation were excluded from the study.

The study was approved by Kaunas Regional Biomedical Research Ethics Committee, No. BE-2–8, issued on 29 October 2014. All patients gave their informed consent.

### 2.2. Transthoracic Echocardiography

Two-dimensional echocardiography was performed using a GE Vivid 7 system (GE Vingmed Ultrasound AS N-3190, Horten, Norway). Echocardiographic studies were performed by an experienced independent echocardiographer, blinded to the patient’s clinical data. Digital loops were stored and analyzed offline (EchoPac V.6.0.0; GE Vingmed).

Anatomic and Doppler examinations and measurements were performed according to American Society of Echocardiography recommendations [11]. The aortic valve area was calculated using the continuity equation utilizing flow velocities in the LV outflow tract and across the valve. A standard evaluation of LV volumes was performed and LV ejection fraction (EF) was calculated according to Simpson’s equation. Early transmitral flow velocity (E) and early diastolic mitral annular velocity (E’) were measured using tissue Doppler in the apical 4-chamber view to provide an estimate of LV diastolic dysfunction (LVDD). The ratio of peak E to peak E’ was calculated (mitral E/E’ ratio) from the average of at least 3 cardiac cycles. The mitral valve deceleration time (MV DT) of the E-wave as well as E/A ratio was also measured. Left atrial (LA) volume was calculated using the biplane area length method at end systole.

Continuous wave Doppler was used to assess maximal tricuspid regurgitation flow velocity to estimate the systolic pressure gradient between the right ventricle (RV) and right atrium (RA). RV systolic pressure was calculated by adding an estimated RA pressure. In our study PH was defined as an estimated PSP ≥ 45 mmHg, based on tricuspid regurgitation flow velocity ≥ 3.0 m/s and presence of other echo PH signs [12].

### 2.3. Blood Sampling

Plasma was sampled 24 h before AVR, processed immediately and NT-proBNP was calculated. Blood samples were stored at −80 °C and GDF-15 was measured with a solid-phase enzyme-linked immunosorbent assay (ELISA) technique. For the evaluation of renal function, serum creatinine levels were determined and glomerular filtration rate was calculated using the CKD-EPI creatinine equation [13].

### 2.4. Statistical Analysis

Continuous variables distribution was verified using the Kolmogorov–Smirnov test. Continuous variables are presented as means (±SD) if normally distributed, and NT-proBNP are expressed as median and interquartile range (because of the very large range of values of the natriuretic peptide and abnormal distribution of this variable). Categorical variables are presented as frequencies and percentages. Characteristics of patients with and without PH were compared using the Student’s t-test for continuous variables and chi-square test for categorical variables. For comparison of two groups the Mann–Whitney test was applied and for three groups the Kruskal–Wallis test. Correlations were computed using Pearson’s method. A two-sided *p* value of <0.05 was used for declaring statistical significance.

Logistic regression analysis was performed using all the independent variables to assess their relative contribution odds ratio (OR) to the development of PSP ≥ 45 mmHg. The incremental value of each clinical and Doppler echocardiography variable in predicting the development of PSP ≥ 45 mmHg was assessed in terms of the construction of receiver-operating characteristic (ROC) curves. All statistical analyses were performed using SPSS 21.0 software (SPSS Inc, Chicago, IL, USA).

## 3. Results

Sixty symptomatic patients (mean age 69 ± 9 years) with severe AS (aortic valve area < 1 cm^2^) were prospectively enrolled in the study. Patients were divided into two groups according to the presence or absence of PH, with PSP cut-off value of 45 mmHg. Thirteen patients (21.7%) had PH (mean systolic PSP 58.5 ± 11.2 mmHg). Clinical, demographic, echocardiographic and biomarker parameters of the overall cohort and those with and without PH characterized by echocardiographic assessment are shown in Table 1.

**Figure 1:**
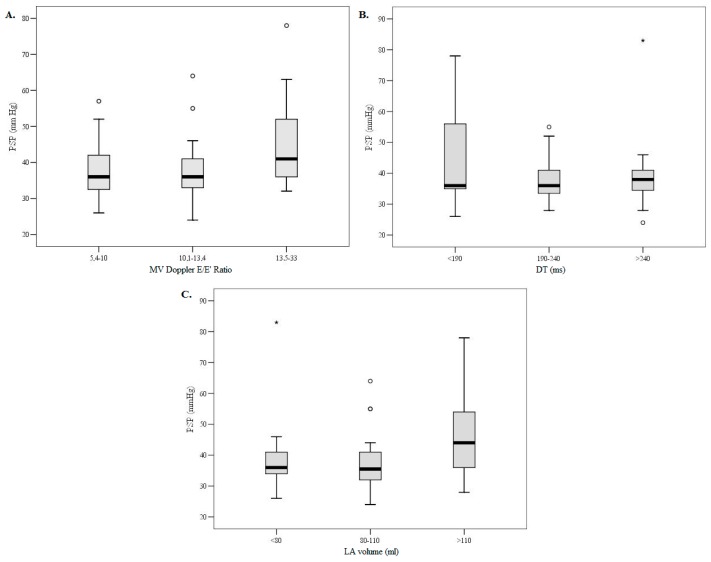
Box-plot of the association of mitral E/E’ ratio (**A**), deceleration time (**B**) and left atrial volume (**C**) by tertiles with PSP. Data are presented as medians (line inside the box), interquartile ranges (IQR) of 25th to 75th percentiles (limits of the box), and 1.5 times IQR above and below the 75th and 25th percentiles (error bars). *p* < 0.05 across tertiles for all variables (E/E’, DT and LA volume). MV = mitral valve; E/E’ = transmitral flow velocity/mitral annular diastolic velocity ratio; DT = deceleration time; LA = left atrium; PSP = pulmonary systolic pressure.

Patients in both groups were similar in terms of age, sex and body mass index (*p* > 0.05). There were no group differences in New York Heart Association (NYHA) functional class and in the prevalence of comorbidities such as arterial hypertension, diabetes mellitus and renal insufficiency (*p* > 0.05). Aortic valve area did not differ between groups; nor did LV EF (50 ± 8 vs 49 ± 8%, *p* = 0.588) and LV EF was not related to elevated PSP. Patients with PH had larger LV dimensions (*p* = 0.05), though LV myocardial mass index did not differ between the groups (*p* = 0.374). Significant differences in parameters of LV diastolic function were found. Increased PSP (≥45 mmHg) was associated with higher E/E’ ratio (*p* = 0.047), shorter MV DT (*p* = 0.014) and larger LA volume (*p* = 0.010). Associations of PSP with parameters of LVDD divided in tertiles, i.e., E/E’ ratio, MV DT and increased LA volume, are presented in Figure 1, respectively.

Biomarker concentrations were higher in patients with aortic stenosis (AS) and elevated PSP (4916 (394–12,032) vs. 602 (366–1818) ng/L, *p* = 0.049 for NT-proBNP and 4525.0 ± 1653.8 vs. 3159.2 ± 1568.7 pg/mL, *p* = 0.030 for GDF-15, respectively). Moderate correlation was found between GDF-15 (*r* = 0.508, *p* = 0.003), NT-proBNP (*r* = 0.496, *p* < 0.001) and elevated PSP. The combination of both biomarkers (GDF-15 and NT-proBNP) strengthened the correlation with PSP (*r* = 0.64, *p* < 0.001).

In univariate logistic regression analysis, LVDD parameters and levels of two biomarkers were highly significant in predicting elevated PSP in severe AS patients. The strongest determinants were E/E’ ratio, MV DT, LA volume, NT-proBNP and GDF-15 values (Table 2). In contrast, age was not an independent determinant of elevated PSP (OR 1.041, *p* = 0.270).

The parameters of worsening LVDD together with elevated levels of two biomarkers were found to be robust predictors of PH in severe AS patients (Table 3).

**Figure 2:**
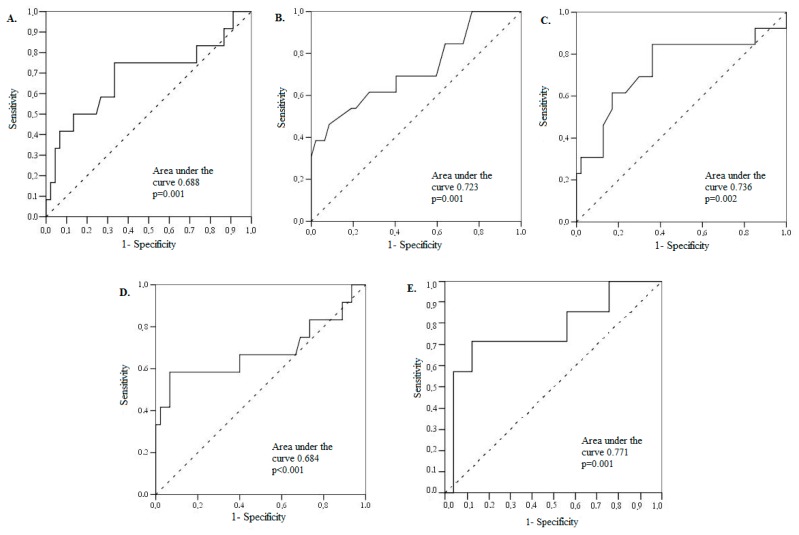
ROC Curve Analysis of E/E’ ratio (**A**), MV deceleration time (**B**), left atrial volume (**C**), NT-proBNP (**D**) and GDF-15 values (**E**). Receiver–operating characteristic (ROC) curve analysis of values of echocardiography-derived E/E’ ratio (**A**), MV deceleration time (**B**), left atrial volume (**C**) and biomarker levels ((NT-proBNP (**D**) and GDF-15 (**E**) in the prediction of PSP ≥ 45 mmHg.

ROC curve analysis of echocardiographic parameters and biomarker levels in prediction of PSP ≥ 45 mmHg is presented in Figure 2.

## 4. Discussion

Our study evaluated the factors for the development of PH in patients with severe AS. The main finding of our data was that concomitant PH in patients with severe AS was associated with worsening of LVDD (higher E/E’ ratio, shorter MV DT and enlargement of LA volume), as well as with elevated levels of biomarkers (NT-proBNP and GDF-15). Several studies reported that in patients with AS the prevalence of PH is high and development of post-capillary PH is significantly associated with worse clinical outcomes and poor prognosis following both transcatheter and surgical AVR [3,5,7,14]. Therefore, to define parameters (clinical, echocardiographic together with biomarkers levels) that are associated with PH in this group of patients is of great importance.

We found that LV diastolic dysfunction, but not LV systolic function, was a predictor of elevated PSP in this group of patients. Several studies concluded that LV systolic function was not associated with PH in AS [15,16], although other data are contradictory [5,17]. All patients in our study had preserved or mid-range LV EF that was not related to elevated PSP. However, LV EF is well known for its lack of sensitivity regarding the detection of subtle myocardial systolic dysfunction [18] and thus seems to be limited in predicting PH in AS patients. Systolic dysfunction is not an early prognostic indicator of PH; it is usually found in the late phase of disease.

Several authors have demonstrated association of PH with LVDD and higher LV filling pressures [15,19]. The degree of PH in AS does not always go hand in hand with the severity of AS, but is strongly associated with left ventricular end diastolic pressure [20]. Patients with AS, who have concomitant moderate or severe mitral regurgitation, can present with increased pressure in the left atrium [21], such patients were excluded from our study. Our study focused on patients who had only mild mitral regurgitation, which was not associated with PH.

LVDD parameters were found to be of great importance for prognosis of increased PSP levels before aortic valve surgery for severe AS. In the present study, worsening of Doppler-derived LVDD parameters (E/E’ ratio > 14, MV DT ≤ 177.5 ms and LA volume > 100 mL) were shown to be independent predictors of PH in severe AS before AVR.

This prospective study demonstrated that not only echocardiographic parameters of LVDD, but also elevated numbers of biomarkers (NT-proBNP and GDF-15) had significant association with PH. Just recently, the measurements of NT-proBNP have been shown to be a useful tool in the identification or exclusion of HF [22]. NT-proBNP is not specific for right- or left-sided heart disease, but circulating levels of this biomarker rise markedly in patients with PH and correlate with severity of disease and mortality [23]. Natriuretic peptides are the only biomarkers that have been widely utilized in patients with AS, although their role in clinical management decisions is not clearly defined. European guidelines indicate that valve replacement may be considered (class IIa recommendation) in patients with asymptomatic severe AS with a “markedly elevated natriuretic peptide level” [24]. The cut-off value of NT-proBNP > 4060 ng/L remained a robust predictor of PH in severe AS patients in this prospective study. The normal range of NT-proBNP (<125 ng/L and <450 ng/L for elderly patients) is inappropriate for this group of patients (almost all had NT-proBNP above this value), because all selected patients had chronically elevated LV filling pressures and half were in NYHA functional class 3–4.

Growth differentiation factor 15 (GDF-15) is a stress-responsive cytokine, the expression of which is strongly up-regulated in cardiac myocytes by various stressors, including increased pressure overload conditions [25]. This biomarker has shown efficacy for assessing outcomes in various cardiovascular diseases, such as heart failure and acute coronary syndromes, as well as in patients undergoing cardiac surgery [26,27,28]. The values of GDF-15 are useful for risk stratification in patients after interventional AVR [8]. In patients undergoing transcatheter AVR, GDF-15 levels were shown to be superior to NT-proBNP for predicting risk [29]. Moderate correlation was found between GDF-15 and PSP. GDF-15 value > 3393 pg/mL was an independent predictor of PH in this study. Either GDF-15 or NT-proBNP alone, or a combination of both biomarkers can improve diagnostic accuracy in patients with AS.

Our findings suggest that in patients with severe isolated AS, LVDD—and not the severity of AS or LV EF—determines PH in symptomatic patients. Further studies are required to assess whether the association of LVDD parameters (E/E’ ratio, MV DT and LA volume) together with biomarkers (GDF-15 and NT-proBNP) can be used to search high risk asymptomatic patients with severe AS and whether these findings could help in the selection of patients, who would benefit from earlier surgery.

## 5. Limitations

A limitation of this study might be a relatively small sample size. Secondly, LV filling pressures and pulmonary pressures were not measured by invasive cardiac catheterization. Although right-heart catheterization (RHC) is the best method to measure the pulmonary artery pressure, echocardiographic estimation of PSP correlates well with RHC measurements [30] and is widely used in the clinical routine.

## 6. Conclusions

Left ventricular diastolic dysfunction and elevated biomarkers levels are factors for the development of pulmonary hypertension in patients with severe aortic stenosis. Elevation of biomarkers paired with worsening of left ventricular diastolic dysfunction could help to stratify patients for earlier surgical treatment before the development of pulmonary hypertension.

## Figures and Tables

**Table 1:** Demographic, clinical, echocardiographic and biomarker parameters of patient cohort.

	Overall (*n* = 60)	PSP < 45 mmHg (*n* = 47)	PSP ≥ 45 mmHg (*n* = 13)	*p* Value
**Age, years**	69 ± 9	68 ± 9	72 ± 10	0.591
**Sex, male *n* (%)**	60 (50)	25 (53.2)	5 (38.5)	0.347
**Glomerulal filtration rate, mL/min/1.73^2^**	84.5 ± 31.4	88.9 ± 33.3	68.5 ± 15.4	0.070
**Body mass index, kg/m^2^**	28.6 ± 4.9	28.8 ± 5.0	27.9 ± 4.5	0.716
**Arterial hypertension, *n* (%)**	48 (80)	38 (80.9)	10 (76.9)	0.754
**Diabetes mellitus, *n* (%)**	5 (8.3)	5 (10.6)	0 (0)	0.219
**NYHA functional class 3–4, *n* (%)**	26 (43.3)	19 (40.5)	7 (53.9)	0.449
**Aortic valve area, cm^2^**	0.81 ± 0.19	0.81 ± 0.19	0.79 ± 0.21	0.829
**Mitral regurgitation mild, *n* (%)**	60 (100)	47 (100)	13 (100)	0.853
**LV end-diastolic diameter index, mm/m^2^**	25.9 ± 3.7	24.1 ± 3.9	27.7 ± 2.8	0.05
**LV Mass index, g/m^2^**	132.2 ± 36.9	129.3 ± 34.9	142.69 ± 43.22	0.374
**LV EF, %**	50 ± 8	50 ± 8	49 ± 8	0.588
**LA volume, mL**	95.4 ± 29.7	90.1 ± 25.7	114.8 ± 35.9	0.010
**PSP, mmHg**	40.5 ± 11.6	35.5 ± 4.8	58.5 ± 11.2	0.001
**E/A ratio**	1.1 ± 0.5	0.9 ± 0.4	1.6 ± 0.8	0.002
**E/E‘ ratio**	14.8 ± 6.3	13.8± 5.4	18.7 ± 8.2	0.047
**MV DT, ms**	226.4 ± 53.9	235.7 ± 50.4	192.7 ± 54.5	0.014
**NT-proBNP, ng/L**	730 (379–2758.5)	602 (366–1818)	4916 (394–12,032)	0.049
**GDF-15, pg/L**	3457.9 ± 662.4	3159.2 ± 1568.7	4525.0 ± 1653.8	0.030

Values are expressed as number (%), mean (±SD) or median (interquartile range); LV = left ventricle; EF = ejection fraction; LA = left atrial; PSP = pulmonary systolic pressure; E = transmitral flow velocity; E’ = mitral annular diastolic velocity; MV = mitral valve; DT = deceleration time; NT-proBNP = N-terminal pro-hormone of brain natriuretic peptide; GDF-15 = growth differentiation factor 15.

**Table 2:** Logistic regression analysis, odds ratio for risk of PSP ≥ 45 mmHg in severe AS.

Parameters/Threshold	β	SE	Chi-Square	OR	95% CI	*p* Value
E/E’ ratio > 14	1.79	0.73	6.74	6.00	1.41–25.48	0.009
DT ≤ 177.5 ms	2.2	0.76	8.76	9.31	2.06–41.14	0.001
LA volume > 100 mL	2.27	0.82	10.23	9.70	1.92–49.03	0.002
NT-proBNP > 4060 ng/L	2.52	0.76	11.86	12.54	2.80–55.99	0.001
GDF-15 > 3393 pg/mL	2.90	1.03	9.26	18.33	2.39–140.39	0.001

SE = standard error; CI = confidence interval; OR = odds ratio; other abbreviations as in Table 1.

**Table 3:** Receiver-operating characteristic (ROC) analysis data. Echocardiographic parameters and biomarker levels in prediction of elevated PSP (≥45 mmHg).

Parameters/Threshold	Area under Curve	95% CI	Sensitivity (%)	Specificity (%)	PPV (%)	NPV (%)	*p* Value
E/E’ ratio > 14	68.8	49.3–88.2	75.0	66.7	37	90	0.009
DT ≤ 177.5 ms	76.2	59.8–92.7	46.2	91.5	39	93	0.001
LA volume > 100 mL	73.6	55.8–91.4	84.6	63.8	60	86	0.002
NT-proBNP > 4060 ng/L	68.4	47.0–89.8	58.3	93.3	70	89	<0.001
GDF-15 > 3393 pg/mL	77.1	54.7–99.6	71.4	88.0	62	92	0.005

NPV = negative predictive value; PPV = positive predictive value; other abbreviations as in Table 2.
